# A permutable MLP-like architecture for disease prediction from gut metagenomic data

**DOI:** 10.1186/s12859-024-05856-w

**Published:** 2024-07-24

**Authors:** Cong Jiang, Jian Yang, Xiaogang Peng, Xiaozheng Li

**Affiliations:** 1 College of Computer Science and Software Engineering, Shenzhen University, Shenzhen, China; 2 National Engineering Laboratory for Big Data System Computing Technology, Shenzhen University, Shenzhen, China; 3 Beijing Key Laboratory of Mental Disorders, National Clinical Research Center for Mental Disorders and National Center for Mental Disorders, Beijing Anding Hospital, Capital Medical University, Beijing, China; 4 Advanced Innovation Center for Human Brain Protection, Capital Medical University, Beijing, China; 5 College of Life Sciences and Oceanography, Shenzhen University, Shenzhen, China; 6 JCY Biotech Ltd., Pingshan Translational Medicine Center, Shenzhen Bay Laboratory, Shenzhen, China

**Keywords:** Metagenomics, Diseases prediction, Deep learning, Permutator, SHAP

## Abstract

Metagenomic data plays a crucial role in analyzing the relationship between microbes and diseases. However, the limited number of samples, high dimensionality, and sparsity of metagenomic data pose significant challenges for the application of deep learning in data classification and prediction. Previous studies have shown that utilizing the phylogenetic tree structure to transform metagenomic abundance data into a 2D matrix input for convolutional neural networks (CNNs) improves classification performance. Inspired by the success of a Permutable MLP-like architecture in visual recognition, we propose Metagenomic Permutator (MetaP), which applied the Permutable MLP-like network structure to capture the phylogenetic information of microbes within the 2D matrix formed by phylogenetic tree. Our experiments demonstrate that our model achieved competitive performance compared to other deep neural networks and traditional machine learning, and has good prospects for multi-classification and large sample sizes. Furthermore, we utilize the SHAP (SHapley Additive exPlanations) method to interpret our model predictions, identifying the microbial features that are associated with diseases.

## Introduction

Metagenomics has made significant advances in the fields of microbial ecology, evolution, and diversity by applying a suite of genomic technologies and bioinformatics tools to directly access the genetic content of entire biological communities over the past two decades [1]. The development of next-generation sequencing technologies has enabled researchers to study the human microbiome, and has made significant progress in the characterization of the microbiome associated with healthy and diseased individuals [2]. In particular, shotgun metagenomic sequencing technology has enabled researchers to obtain a higher resolution profile of the microbial community at the species- and strain-levels [3, 4]. Extensive metagenomic studies have shown that the gut microbiota is closely associated with various host diseases, including obesity [5], type 2 diabetes [6], cirrhosis [7], etc. [8].

There are trillions of microbes inhabiting the human intestine, forming a complex and stable ecosystem that is closely related to human health [9]. The characteristics of a metagenomic sample are commonly described by the relative abundance of microbial taxa at one of the taxonomic levels(i.e. Phylum, Class, Order, Family, Genus, and Species) [10]. Although next-generation sequencing technologies have greatly enhanced our capacity to characterize the metagenomic data, we still face challenges related to high dimensionality, limited sample sizes, and sparse features in metagenomic data.

In the past few years, disease prediction models based on metagenomic data can be broadly categorized into two major types: 1) traditional machine learning-based prediction models, and 2) deep learning-based prediction models. Traditional machine learning models are commonly used for metagenomic data include Random Forest (RF), Least Absolute Shrinkage and Selection Operator (LASSO), and Support Vector Machines (SVM) [11–13]. These methods utilize unordered vectors of species-level relative abundance or strain-specific markers for performing supervised classification learning. However, this high-dimensional and sparse tabular data is not the optimal data format for achieving efficient deep learning [14].

One distinctive characteristic of metagenomic data is the phylogenetic tree that establishes connections among microbial species, with closely related species exhibiting similar relationships in the environment and disease [15]. Therefore, the input features of deep learning models typically integrate phylogenetic information to transform raw data into “synthetic images” or 2D matrices. Recent studies have demonstrated that transforming raw metagenomic features into feature representations that incorporate phylogenetic tree information can significantly enhance the classification performance of deep learning models [10, 15–17]. Marculescu et al. [16] and Nguyen et al. [17] introduced a phylogenetic-sorting method that enables effective exploration of convolutional neural networks for disease classification. The phylogenetic-sorting approach involves extracting phylogenetic information by sorting the microbial features based on their taxonomic annotation in alphabetical order, by concatenating the strings of their taxonomy [16, 17]. However, this approach disregards the relationships between species sharing the same ancestor but having greater alphabetical distances in their names. Reiman et al. [10] and Zhu et al. [15] respectively developed PopPhy-CNN and Cascade Deep Forest, which were trained using 2D matrices incorporating embedded phylogenetic tree information as input data. However, their embedding approach [10, 15] uses the 2D matrix that includes all nodes of the phylogenetic tree, resulting in sparsity in the upper part of the matrix due to the branching and hierarchical structure of the tree.

In this study, we addressed the challenge of data sparsity in the matrix when transforming raw metagenomic features into matrix representations that incorporate phylogenetic tree information, achieved by reducing the matrix height to solely focus on phylogenetic structure information derived from leaf nodes. This strategy aims to preserve the integrity of the phylogenetic tree structure while enhancing computational efficiency, resulting in a denser representation in the form of the 2D matrix. Moreover, by drawing inspiration from the successful utilization of a Permutable MLP-like architecture in the field of image classification [18], we introduced MetaP, the Permutable MLP-like architecture for the classification of metagenomic data that incorporates the embedded phylogenetic information. We demonstrated the competitive performance and robustness of MetaP in both binary and multiclass classification tasks through benchmark testing with several well-established machine learning methods (RF, SVM) [11] and other deep learning methods (MetaNN [16], PopPhy-CNN [10]) on three publicly available datasets. Finally, we incorporated the SHAP [19] method for interpretable analysis of our model predictions, aiming to uncover the associations between the identified crucial microbes and specific diseases.

## Materials and methods

**Fig. 1:**
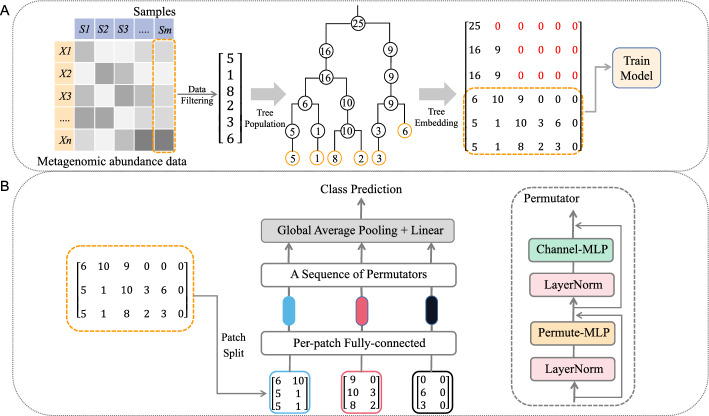
Overview of MetaP model. **A** Example of populating and embedding phylogenetic tree. We only consider embedding the phylogenetic structure information derived from the leaf nodes of the phylogenetic tree into the matrix, thereby avoiding data sparsity within the matrix. **B** Basic architecture of the proposed MetaP. We split the matrix into fixed-size patches, linearly embed each of them, and then feed them into a sequence of standard Permutators [18] for feature encoding. A global average pooling layer followed by a fully-connected layer is finally used to predict the class

In this section, we outline the key components as depicted in Fig. 1. Additionally, we discuss the application of the SHAP method in interpreting model outputs and provide an overview of the dataset source.

### Representing metagenomic data using 2D matrices

The process of representing metagenomic abundance data using 2D matrices based on the phylogenetic tree, similar to PopPhy [20], is depicted in Fig. 1A. We utilized PhyloT [21] to generate phylogenetic trees, assuming a constant distance of one between nodes in the tree. Each feature in the metagenomic samples is referred to as an Operational Taxonomic Unit (OTU), and we use species-level relative abundances obtained from metagenomic sequencing as the values for these OTUs. For each raw metagenomic dataset, we first filter out OTUs that exhibit a prevalence lower than the threshold of 10% in all classes, and then generate a corresponding pruned phylogenetic tree based on the observed OTUs. This tree serves as a template to construct a populated tree for each sample in the dataset. The leaf nodes are then populated with their corresponding OTU abundance values, while the abundance of each non-leaf node is determined by summing the abundances of its child nodes. The 2D matrix representation is generated by performing a level-order traversal of the phylogenetic tree, populating the matrix in a top-to-bottom and left-to-right order, and assigning zeros to empty positions without any nodes. However, since the OTU value represents the species-level relative abundance within the sample, ranging from 0 to 100, and with the cumulative sum of each sample equating to 100, employing the aforementioned method to populate the node values of parent nodes as the sum of their respective child node values leads to diminished discriminability in the upper portion of the tree. Additionally, the branching and hierarchical structure of the tree also result in sparsity in the upper part of the matrix. This prompts us to reduce the matrix height to include only the phylogenetic structure information derived from the leaf nodes, allowing us to obtain a more compact and efficient matrix representation of the data.

### A permutable MLP-like architecture

The Permutable MLP-like architecture [18], proposed by Hou et al., is a conceptually simple and data efficient MLP-like architecture designed for visual recognition. In this study, we explore the application of this architecture in the classification of metagenomic data. The basic architecture of the Permutable MLP-like networks can be found in Fig. 1B. Our network takes a 2D matrix as input and uniformly splits it into a sequence of non-overlapping matrix patches. Each patch is mapped into a linear embedding (or called token), using the same projection matrix. These tokens are subsequently fed into a sequence of Permutators to encode both spatial and channel information. The resulting tokens are finally processed using global average pooling followed by a linear classifier to make class predictions.

**Fig. 2:**
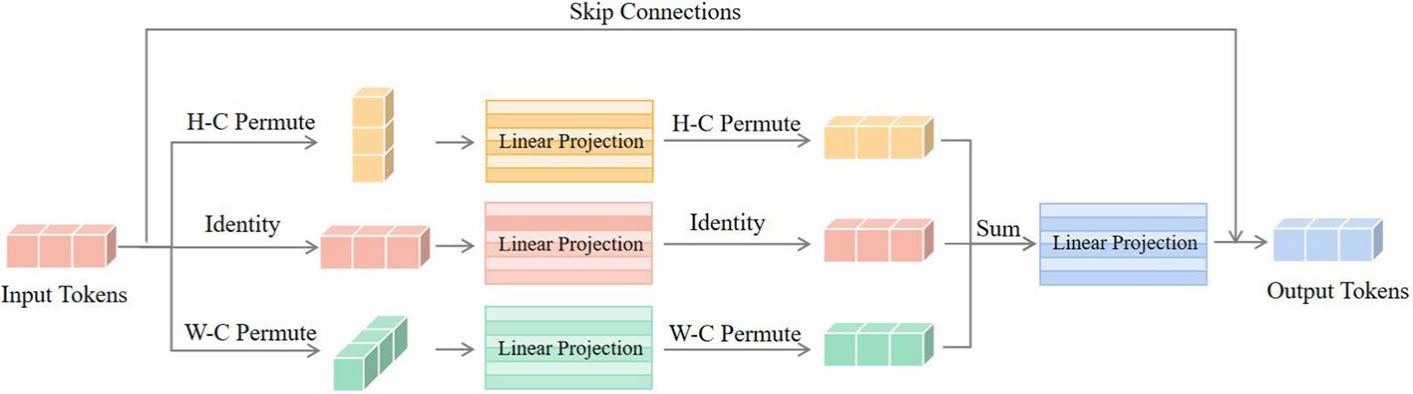
Basic structure of the Permute-MLP layer. The Permute-MLP layer contains three branches dedicated to encoding features across the dimensions of height, width, and channel. Subsequently, the outputs from these branches are combined through element-wise addition, followed by a fully-connected layer for feature fusion

As the bottom-right corner of Fig. 1B illustrates, in order to learn the spatial and channel information of the matrix, Hou et al. [18] introduces the Permutator block, which consists of two components: Permute-MLP and Channel-MLP. As shown in Fig. 2, The Permute-MLP module accepts 3-dimensional token representations and consists of three branches, each responsible for encoding information along the height, width, or channel dimension [18]. The Channel-MLP module shares a similar structure to the feed forward layer in Transformers [22] that comprises two fully-connected layers with a GELU activation in the middle [18]. Mathematically, given an input C-dim tokens $X \in \mathbb {R}^{H \times W \times C}$, the formulation of Permutator can be written as follows:

1 $$\begin{aligned} \textbf{Y}= & {} {\text {Permute-MLP}}({\text {LN}}(\textbf{X})) + \textbf{X}, \end{aligned}$$

2 $$\begin{aligned} \textbf{Z}= & {} {\text {Channel-MLP}}({\text {LN}}(\textbf{Y})) + \textbf{Y}, \end{aligned}$$

where, LN refers to LayerNorm [18]. The output Z will serve as the input to the next Permutator block until the last one [18].

### Interpreting model predictions using SHAP

Shapley Additive Explanations (SHAP) [19], proposed by Lundberg and Lee in 2017, is a method for interpreting the outputs of any complex models. This method is grounded in a solid theoretical framework derived from game theory, aiming to estimate SHAP values, which represent the contribution of each input feature to the final model prediction. In our experiment, we employed the Kernel SHAP Explainer module(available at https://github.com/slundberg/shap), which utilizes a specially-weighted local linear regression approach to estimate SHAP values. This method relies on a background dataset for training and simulates feature absence by substituting the feature with the values it takes in the background dataset. The importance of each feature in the kernel Shapley value is determined by comparing the prediction value obtained with the feature to the prediction value obtained without it. Positive SHAP values suggest that a feature has a positive impact on the model’s prediction, while negative values suggest a negative impact. The magnitude of the value indicates the strength of the contribution, with larger magnitudes indicating greater influence. In our experiment, to obtain the SHAP values corresponding to each feature, we performed a 10-fold cross-validation and computed the average of the SHAP values obtained from each fold. During each fold of training, due to computational resource limitations, we utilized the k-means [23] function to summarize the training dataset as the background dataset for Kernel SHAP, and then calculated the average SHAP value for all features of the samples in the test set.

### Datasets

In this study, we select publicly available datasets from the MetAML package [11]: Cirrhosis, Type 2 Diabetes (T2D), and Obesity datasets. Table 1 provides a summary of these datasets. The Cirrhosis dataset comprises 114 cirrhotic patients and 118 healthy individuals. The T2D dataset consists of 170 patients with type 2 diabetes and 174 instances of control samples. The Obesity dataset consists of 253 samples, where 89 samples have a BMI lower than $25\,{\text {kg/m}}^2$, while the remaining 164 samples have a BMI greater than $30\,{\text {kg/m}}^2$. In the MetAML study [11], all data were obtained through metagenomic shotgun sequencing [24], and the OTUs for each sample were obtained using MetaPhlAn2 [25] with default parameters. In our study, the OTUs are aggregated at the species level within each dataset. For any OTU that is classified as “unclassified” at the species level, the label of the next highest taxonomic level is assigned to that OTU.

**Table 1:** Summary of the datasets used in the experiments

Dataset	# Total samples	# Case samples	# Control samples	# Features
Cirrhosis	232	114	118	542
T2D	344	170	174	572
Obesity	253	164	89	465

## Results and discussion

In this section, we will provide an exposition of the experimental setup and subsequently delve into an analysis and discussion of the experimental results.

**Table 2:** Classification performance comparison of MetaP with existing methods

DataSet	Metric	MetAML		MetaNN		PopPhy-CNN	MetaP
		RF	SVM	MLPNN	1DCNN		
Cirrhosis	AUC	**0.947 (0.045)**	0.934 (0.053)	0.896 (0.063)	0.881 (0.066)	0.922 (0.062)	0.942 (0.049)
	MCC	**0.759 (0.119)**	0.718 (0.129)	0.638 (0.151)	0.589 (0.165)	0.719 (0.142)	0.749 (0.124)
	Precision	**0.884 (0.058)**	0.864 (0.064)	0.824 (0.076)	0.801 (0.082)	0.865 (0.069)	0.879 (0.060)
	Recall	**0.875 (0.061)**	0.855 (0.065)	0.815 (0.075)	0.787 (0.084)	0.854 (0.073)	0.870 (0.065)
	F1	**0.874 (0.061)**	0.853 (0.066)	0.813 (0.076)	0.784 (0.086)	0.852 (0.074)	0.869 (0.066)
T2D	AUC	0.746 (0.075)	0.584 (0.177)	0.680 (0.079)	0.641 (0.073)	0.721 (0.076)	**0.784 (0.071)**
	MCC	0.335 (0.144)	0.193 (0.186)	0.252 (0.153)	0.189 (0.129)	0.304 (0.161)	**0.448 (0.134)**
	Precision	0.669 (0.072)	0.534 (0.186)	0.628 (0.078)	0.597 (0.066)	0.654 (0.082)	**0.727 (0.068)**
	Recall	0.665 (0.071)	0.594 (0.091)	0.624 (0.074)	0.592 (0.063)	0.649 (0.079)	**0.721 (0.067)**
	F1	0.663 (0.072)	0.546 (0.149)	0.620 (0.076)	0.586 (0.064)	0.646 (0.080)	**0.719 (0.067)**
Obesity	AUC	0.652 (0.098)	0.626 (0.107)	0.593 (0.115)	0.543 (0.124)	0.622 (0.110)	**0.661 (0.108)**
	MCC	0.039 (0.169)	0.037 (0.174)	0.141 (0.190)	0.043 (0.205)	0.161 (0.213)	**0.166 (0.189)**
	Precision	0.549 (0.145)	0.533 (0.141)	0.611 (0.089)	0.562 (0.124)	0.620 (0.100)	**0.627 (0.097)**
	Recall	0.640 (0.041)	0.631 (0.056)	0.614 (0.088)	0.614 (0.077)	0.605 (0.098)	**0.644 (0.071)**
	F1	0.542 (0.053)	0.544 (0.065)	0.602 (0.086)	0.561 (0.080)	0.604 (0.098)	**0.618 (0.078)**
Multi-disease	AUC	–	–	–	–	–	–
	MCC	0.542 (0.062)	0.411 (0.073)	0.437 (0.074)	0.438 (0.085)	0.518 (0.067)	**0.558 (0.063)**
	Precision	**0.711 (0.044)**	0.622 (0.049)	0.620 (0.050)	0.632 (0.058)	0.662 (0.053)	0.699 (0.044)
	Recall	0.687 (0.040)	0.607 (0.046)	0.614 (0.048)	0.619 (0.055)	0.644 (0.051)	**0.693 (0.043)**
	F1	0.678 (0.042)	0.596 (0.048)	0.610 (0.050)	0.613 (0.057)	0.637 (0.055)	**0.691 (0.044)**

Mean AUC, MCC, Precision, Recall, and F1 scores from 10-fold cross-validation (repeated 10 times) are reported. Best values are highlighted in bold, standard deviation is shown in parentheses

### Experimental setup

The Permutable MLP-like network structure parameter settings for our model applied to metagenomic classification are described as follows. Due to the requirement of ensuring divisibility between the patch dimensions and the 2D matrix, we implemented specific parameter settings. Specifically, the patch width was set to 3, and during the construction of the 2D matrix, we ensured that its width was divisible by 3. Furthermore, the patch height was set to match the reduced matrix height, which typically does not exceed 8. For example, in the T2D dataset, the matrix height was only 5.

There are several hyper parameters in MetaP, including the number of Permutator blocks $P_n$, the channel dimension split segments $S_n$, the hidden units in the neural network $h_u$, the initial learning rate *lr* of the Adam [26] optimizer, the batch size *bs*, the number of iterations *epoch*, and two parameters (step size $\alpha$ and decay factor $\gamma$) associated with the Step learning rate scheduler (StepLR) provided by PyTorch for learning rate scheduling. These parameters are explored over different combinations from the following ranges: $P_n \in \{1, 2, 3\}$, $S_n \in \{2, 4, 6, 8, 10\}$, $h_u \in \{32, 48, 64, 128\}$, $lr \in \{0.05, 0.005, 0.0005, 0.00005\}$, $bs \in \{16, 32, 64, 128\}$, $epoch \in \{10, 20, 30, 40, 50\}$, $\alpha \in \{5, 10\}$, $\gamma \in \{0.1, 0.3, 0.5, 0.7\}$. After adjustments, we identified the optimal parameters for MetaP in subsequent experiments as $P_n=1, S_n=8, h_u=48, lr=0.0005, bs=32, epoch=20, \alpha =5, \gamma =0.5$.

In order to verify the effectiveness of our proposed method, we compared it with the MetAML [11], MetaNN [16], and PopPhy-CNN [10] methods proposed in previous studies. Besides, for the other comparison models, we followed the recommended hyperparameter settings and model architectures outlined in their respective original papers or the provided open-source code. In our experiment, we performed 10-fold cross-validation 10 times and then computed the average to ensure the accuracy of our results. We employed five metrics, namely Area under curve-receiver operating characteristic (AUC), Matthews correlation coefficient (MCC), Precision, Recall, and F1 score, to comprehensively evaluate the model from different perspectives.

We separately evaluated the performance of our model in binary and multiclass classification tasks. For binary classification task, we conducted experiments on the three publicly available datasets mentioned in previous sections. For multiclass classification task, we created a Multi-Disease dataset consisting of 829 samples with multiple diseases by combining these three datasets based on species-level abundance features present in each sample.

**Fig. 3:**
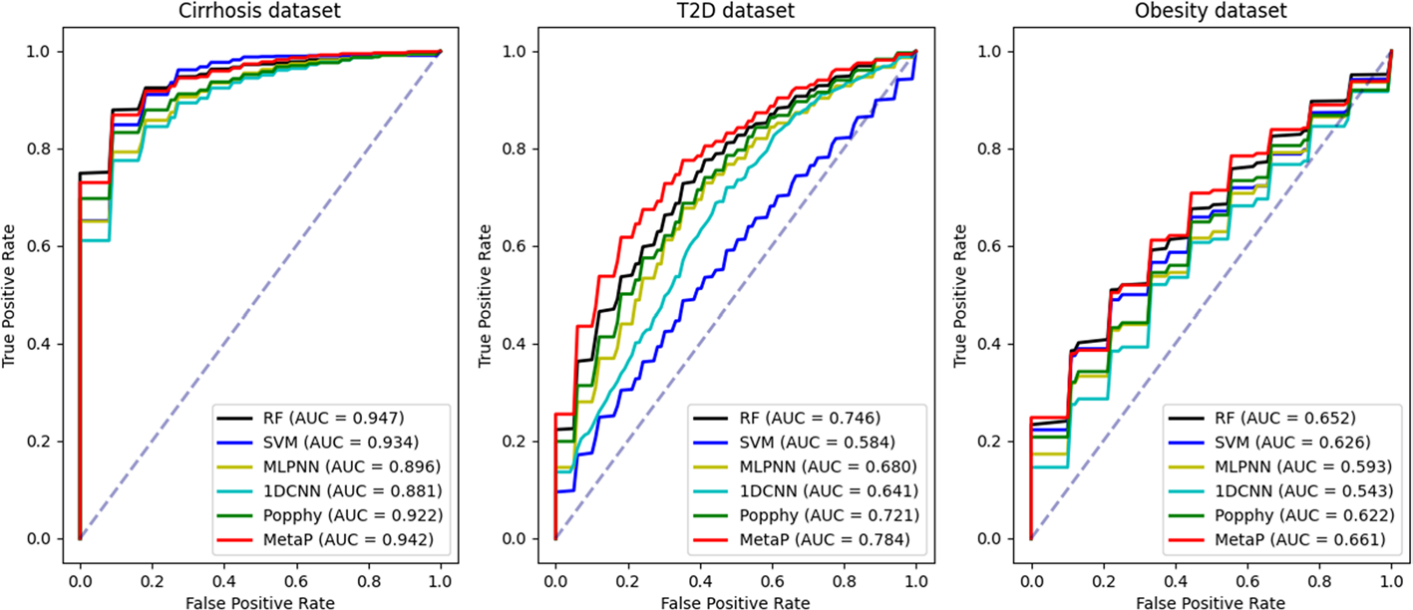
Comparing the ROC curves of MetaP and other five methods based on 10-fold cross-validation on the three disease datasets

**Table 3:** Comparison of PopPhy-CNN and MetaP on dense versus sparse matrix classification performance

DataSet	Metric	PopPhy-CNN			MetaP		
		Sparse	Dense	Difference	Sparse	Dense	Difference
Cirrhosis	AUC	0.922 (0.062)	0.928 (0.048)	+0.006	0.934 (0.050)	0.942 (0.049)	+0.008
	MCC	0.719 (0.142)	0.731 (0.131)	+0.012	0.744 (0.153)	0.749 (0.124)	+0.005
	Precision	0.865 (0.069)	0.872 (0.064)	+0.007	0.877 (0.075)	0.879 (0.060)	+0.002
	Recall	0.854 (0.073)	0.859 (0.067)	+0.005	0.867 (0.078)	0.870 (0.065)	+0.003
	F1	0.852 (0.074)	0.857 (0.068)	+0.005	0.866 (0.079)	0.869 (0.066)	+0.003
T2D	AUC	0.721 (0.076)	0.725 (0.083)	+0.004	0.790 (0.070)	0.784 (0.071)	−0.006
	MCC	0.304 (0.161)	0.324 (0.149)	+0.020	0.425 (0.144)	0.448 (0.134)	+0.023
	Precision	0.654 (0.082)	0.665 (0.077)	+0.011	0.715 (0.074)	0.727 (0.068)	+0.012
	Recall	0.649 (0.079)	0.659 (0.073)	+0.010	0.710 (0.071)	0.721 (0.067)	+0.011
	F1	0.646 (0.080)	0.655 (0.074)	+0.009	0.708 (0.071)	0.719 (0.067)	+0.011
Obesity	AUC	0.622 (0.110)	0.625 (0.110)	+0.003	0.667 (0.110)	0.661 (0.108)	−0.006
	MCC	0.161 (0.213)	0.139 (0.193)	−0.022	0.155 (0.199)	0.166 (0.189)	+0.011
	Precision	0.620 (0.100)	0.609 (0.092)	−0.011	0.616 (0.103)	0.627 (0.097)	+0.011
	Recall	0.605 (0.098)	0.595 (0.089)	−0.010	0.643 (0.074)	0.644 (0.071)	+0.001
	F1	0.604 (0.098)	0.593 (0.089)	−0.011	0.615 (0.085)	0.618 (0.078)	+0.003
Multi-disease	AUC	–	–	–	–	–	–
	MCC	0.518 (0.067)	0.527 (0.068)	+0.009	0.554 (0.060)	0.558 (0.063)	+0.004
	Precision	0.662 (0.053)	0.668 (0.053)	+0.006	0.696 (0.042)	0.699 (0.044)	+0.003
	Recall	0.644 (0.051)	0.653 (0.050)	+0.009	0.689 (0.041)	0.693 (0.043)	+0.004
	F1	0.637 (0.055)	0.646 (0.053)	+0.011	0.686 (0.041)	0.691 (0.044)	+0.005

We label the matrix without height reduction as “Sparse” and the matrix with height reduction as “Dense”. The column header “Difference” represents the disparity in classification performance of the same model trained on dense matrix compared to sparse matrix. Mean AUC, MCC, Precision, Recall, and F1 scores from 10-fold cross-validation (repeated 10 times) are shown. Standard deviation is shown in parentheses

### The classification performance of models

The classification performance results of different models on the dataset are presented in Table 2, and for better visual comparison, the corresponding ROC curves of RF, SVM, MLPNN, 1DCNN, PopPhy-CNN, and MetaP are shown in Fig. 3. Among the machine learning algorithms, Random Forest(RF) exhibits the best classification performance in the metagenomic datasets. Our experimental results demonstrate that our proposed MetaP model achieved the best classification performance on the Obesity, T2D, and Multi-Disease datasets, while exhibiting slightly lower performance compared to RF on the Cirrhosis dataset. We attribute the superior classification performance of our MetaP model on these datasets to their larger sample size compared to the Cirrhosis dataset. Previous studies [27] have also demonstrated that deep learning models generally outperform traditional machine learning methods when applied to datasets with a larger number of samples.

Additionally, we trained the PopPhy-CNN and MetaP models on both sparse and dense matrices. Our experimental results, as shown in Table 3, demonstrate that in most cases, reducing the matrix height to mitigate data sparsity does not have an adverse impact on the model’s classification performance. On the contrary, the model’s classification performance exhibits a slight improvement, which may be attributed to the enhanced focus on important features and the reduction of interference from redundant information. However, the PopPhy-CNN model using convolutional neural network experiences a slight decline in classification performance for the Obesity dataset with fewer features, whereas our proposed MetaP model remains unaffected. Overall, we believe that reducing the matrix height in this manner is effective in improving computational efficiency without compromising classification performance.

### Identification of disease-associated microbial features

**Fig. 4:**
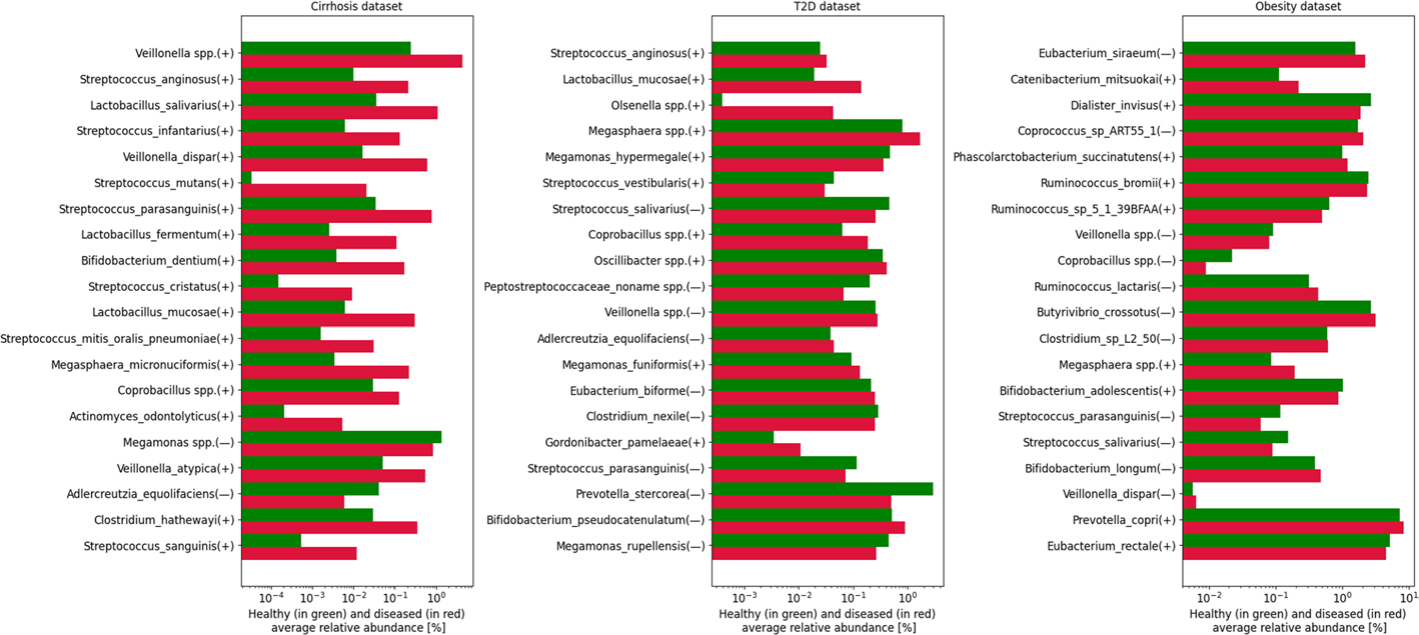
The top 20 microbial features with the highest SHAP values in each of the three disease datasets. For each species, the SHAP values on the vertical axis decrease from top to bottom, while the two horizontal bars represent the average relative abundance observed in the healthy samples (depicted in green) and the diseased samples (depicted in red). The positive or negative signs inside the parentheses indicate the positive or negative values of the SHAP values

To interpret the black box nature of the MetaP model and identify the microbial features that play a significant role in classification, we utilized the Kernel SHAP method on our model to obtain the SHAP values corresponding to each feature from a 10-fold cross-validation. In Fig. 4, we present the top 20 important microbial features based on the SHAP values in each of the three disease datasets, along with their average relative abundances in the samples. Similarly to previous studies [11], We found that the importance of each microbial feature was not strongly correlated with its average relative abundance across samples, indicating the complexity of the microbial system.

In the Cirrhosis dataset, which exhibited the best performance in terms of model classification, our model identified the following important microbial features: the Veillonella, Lactobacillus, and Streptococcus genera. These important features were also identified in the original study [7, 28]. In the T2D and Obesity datasets, which exhibited lower classification accuracy, the important microbes identified by our model can serve as candidate sets for future experimental studies investigating the association between microbes and diseases. And our model also detected important species that have been previously reported in the literature. For example, associations between specific species such as *Lactobacillus mucosae* [29] and *Olsenella* spp. [30] with T2D patients, as well as associations between species like *Ruminococcus bromii* [31] and *Eubacterium siraeum* [32] with Obesity patients were identified. Additionally, we observed a correlation between the positive/negative SHAP values and the differences in the average abundance of microbes between the patients and healthy individuals, particularly in datasets with good classification performance. Microbes that have a higher relative abundance in the diseased population often exhibit positive SHAP values. For instance, in the Cirrhosis dataset, among the top 20 important microbes, only Megamonas spp. and Adlercreutzia equolifaciens showed higher abundance in the healthy population with negative SHAP values, while the remaining microbes exhibited higher relative abundance in the diseased population. In conclusion, we believe that improving the classification performance of models on metagenomic data can unveil potential associations between microbes and diseases, warranting further investigation.

## Conclusion

We applied a Permutable MLP-like architecture for disease prediction from metagenomic data. This network structure, compared to deep neural networks, allowed us to effectively capture the phylogenetic information of microbes within the 2D matrix formed by embedding the phylogenetic tree. Additionally, we improved the embedding method of the phylogenetic tree based on PopPhy into the matrix by reducing the matrix height to accommodate only the phylogenetic structure information derived from the leaf nodes, thereby mitigating data sparsity and redundant information. We evaluated the performance of our model on three publicly available datasets and compared its effectiveness with established methods such as MetAML, MetaNN, and PopPhy-CNN, for both binary and multi-class classification tasks. Lastly, we presented the interpretability of our model’s predictions by incorporating the SHAP method.

We believe that our proposed MetaP model has contributed to the advancement of deep learning in the field of gut microbiome research, enabling the exploration of potential associations between gut microbiome and human diseases. Moreover, we found that deep learning models are better suited for large-scale sample datasets, indicating that data augmentation for metagenomic data could be a promising research direction. In recent years, both metagenomics research and deep learning techniques have been rapidly advancing, it is imperative for us to actively explore the rational integration of these two fields, leveraging the research achievements in computer science to propel the development of metagenomic data analysis.

## Data Availability

The datasets and the code used in this study can be found at https://github.com/ansjiang/MetaP.
